# Hairy cell leukemia expresses programmed death-1

**DOI:** 10.1038/s41408-020-00384-1

**Published:** 2020-11-05

**Authors:** Priyadarshini Kumar, Qi Gao, Alexander Chan, Natasha Lewis, Allison Sigler, Janine Pichardo, Wenbin Xiao, Mikhail Roshal, Ahmet Dogan

**Affiliations:** Hematopathology Service, Department of Pathology, Memorial Sloan Kettering Cancer Center, 1275 York Ave, New York, NY 10065 USA

**Keywords:** Tumour immunology, Hairy cell leukaemia

Programmed death-1 (PD-1) is a lymphoid receptor that negatively regulates immune responses^1^. Immune checkpoint inhibitors, including PD-1 and its ligand (PD-L1) inhibitors, have shown promise in treating several types of malignancies, including melanoma, classical Hodgkin lymphoma (CHL), non-small cell carcinoma of the lung, kidney, bladder, and head and neck cancers^2,3^. PD-1 is mainly expressed in reactive and neoplastic T cells, for example, angioimmunoblastic T cell lymphoma, but reports of expression were also noted in naive and activated B cells as well as neoplastic B cells including chronic lymphocytic leukemia/small lymphocytic lymphoma (CLL/SLL), high-grade follicular lymphoma (FL), and diffuse large cell B-cell lymphoma^4^. In contrast, other B-cell lymphomas such as, mantle cell (MCL), marginal zone (MZL), Burkitt, and low-grade FL are PD-1 negative^5^. In CLL/SLL, PD-1 is overexpressed on the neoplastic B cells in comparison to peripheral blood B cells of healthy volunteers, furthermore, expression of PD-1 was higher in cases with mutated immunoglobulin heavy chain variable (*IGHV*) genes^6^. Previous studies that have sought to characterize PD-1 expression in B-cell neoplasms primarily using immunohistochemical analysis and therefore a more extensive study of PD-1 expression in leukemic B-cell neoplasms, particularly those with a post-germinal center, or mutated *IGHV* including hairy cell leukemia (HCL) has not been performed.

To investigate further we systematically analyzed PD-1 expression in low-grade B-cell neoplasms using a clinical triage flow cytometry assay in peripheral blood, bone marrow aspirates, lymph nodes, body fluids, and other tissues (Fig. 1A). Final pathologic diagnoses were established according to the criteria of the 2016 World Health Organization classification using morphology, immunohistochemical staining, and cytogenetic analysis, where applicable^7^.

**Fig. 1:**
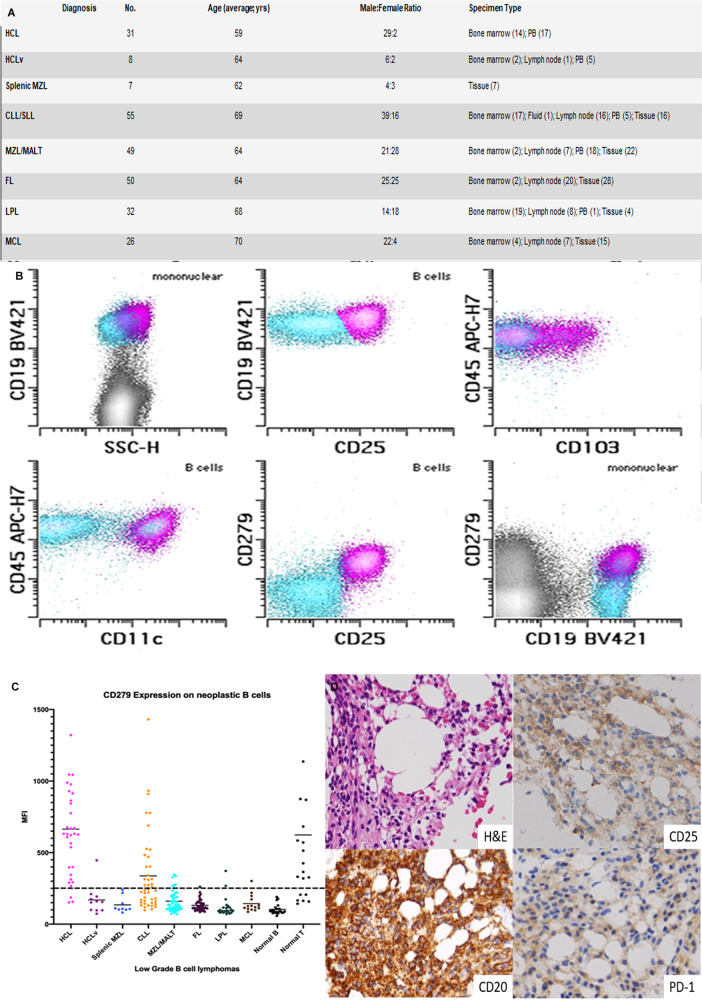
Expression of PD-1 in B-cell lymphoproliferative disorders. **A** Characteristics of B-cell lymphoproliferative disorders included in the study. HCL, hairy cell leukemia; HCLv, hairy cell leukemia-variant; MZL, marginal zone lymphoma; CLL/SLL, chronic lymphocytic leukemia/small lymphocytic lymphoma; MZL/MALT, marginal zone lymphoma (includes extranodal marginal zone lymphoma of mucosa-associated lymphoid tissue (MALT)); FL, follicular lymphoma; LPL, lymphoplasmacytic lymphoma; MCL, mantle cell lymphoma. **B** CD19^+^ B cells from a case of hairy cell lymphoma, showing aberrant expression of CD25, CD103 (partial), CD11c, and CD279 (pink), normal B cells (blue). **C** CD279 (PD-1) expression in low-grade B-cell lymphoproliferative disorders. Mean fluorescence intensities (MFIs) and standard deviation of PD-1 expression are shown for a range of B-cell lymphoproliferative disorders. Greater than 260 MFI was considered positive for CD279. Most cases of hairy cell leukemia (HCL) were positive for CD279 (664.5 ± 370.7 (151.9–1901)). CLL/SLL (337.4 ± 277.9 (97.94–1431)), MZL/MALT (160.4 ± 71.91 (70.04–345.7)), HCLv (169.0 ± 102.0 (73.01–373.3)), splenic MZL (134.7 ± 56.41 (81.58–158.3)), LPL (121.0 ± 72.16 (70.97–372.0)), FL (131.9 ± 38.59 (84.51–177.1)), MCL (143.7 ± 59.70 (86.38–215.5)), normal B (103.4 ± 35.32 (58.02–189.7)), normal T (623.5 ± 614.5 (142.9–2455)). **D** Immunohistochemistry on a select bone marrow specimen with extensive involvement by HCL (H&E); CD20 is positive with co-expression of CD25 (positive) and PD-1 (positive).

To investigate the expression of PD-1 on the B cells, 10-color immunophenotypic analysis was performed using a FACS Canto 10 cytometer (BD Biosciences, San Jose CA). Flow cytometric data were analyzed with Woodlist software (Wood BL, University of Washington). Lymphocytes were first gated using the forward vs side scatter. Relevant neoplastic populations were then specifically gated based on CD45 and CD19 expression, and finally any abnormal antigen expression. Mean fluorescence intensity (MFI) of the neoplastic population in the CD279 (PD-1/Clone: EH12.1) BV605 channel was determined using the statistics options available in Woodlist. In a subset of cases, to confirm that CD279 was specifically on neoplastic hairy cells, an additional tube was performed and the presence of CD279 was identified in all samples tested (Fig. 1B). Statistical analysis was performed using GraphPad Prism 8 software (GraphPad Software, La Jolla, CA).

A total of 219 specimens of bone marrow, lymph nodes, peripheral blood, body fluids and other tissues known to be involved by a B-cell lymphoproliferative disorder were analyzed for CD279 expression, along with other markers. Findings were correlated with morphologic, immunophenotypic, cytogenetic, and molecular findings to verify the final diagnosis. HCL patients presented with cytopenias and/or splenomegaly and bone marrow involvement by a clonal population of intermediate-sized lymphocytes with cytoplasmic projections. A *BRAF* or *MAP2K* pathway mutation was found in 31/32 cases. There were 11 cases of B-cell lymphoproliferative disorders with the characteristic morphologic appearance and partial aberrant immunophenotype, with negative *BRAF* and/or *MAP2K* pathway mutations, classified here as hairy cell leukemia-variant (HCLv), although the differential includes HCLv, splenic diffuse red pulp, and splenic lymphoma, unclassifiable. There were 9 cases of splenic marginal zone lymphoma (MZL), who presented with splenic involvement and/or del 7q with villous cytoplasmic protrusions.

In addition to CLL/SLL, we identified that HCL expresses high levels of PD-1 when compared to other lymphoproliferative disorders, including HCLv (Fig. 1C). There were 32 cases of HCL studied by flow cytometric immunophenotypic analysis from patients described in Fig. 1A. Most cases of HCL exhibited very high levels of CD279 expression, whereas HCLv, MZL/MALT, and splenic MZL were typically negative. Follicular lymphoma (FL), lymphoplasmacytic lymphoma (LPL), and mantle cell lymphoma (MCL) showed variable low-level expression. HCL exhibited a high level of CD279 expression compared with non-neoplastic B cells (negative control) and a similar level of expression compared to reactive non-neoplastic T cells (positive control). When comparing the average MFIs of the positive and negative controls, we considered a cutoff of 260. ROC curve analysis identified a CD279 MFI threshold (>371.2) for which the diagnosis of HCL could be assessed with 75% sensitivity and 98.5% specificity (Supplemental Data) using a combination of HCLv, splenic MZL, and MZL as controls.

We found that the expression level of CD279 by HCL was higher than in CLL/SLL and similar to that of “exhausted” T cells in normal flow specimens. The differences in CD279 MFI results for HCL and other low-grade B-cell lymphomas were statistically significant; *P* < 0.0001 for HCL vs. HCLv, HCL vs. Splenic MZL, HCL vs MZL, HCL vs. FL, HCL vs. CLL/SLL, etc. To investigate whether the sample type created a statistically significant difference, we examined PD-1 staining in 10 normal PB samples, 10 bone marrow aspirate samples, and 10 normal lymph node tissue samples. No statistically significant difference was identified. Immunohistochemistry for PD-1 and PD-L1 was also performed on bone marrow biopsies, where the involvement of the HCL exceeded 40% of the biopsy. PD-1 staining was positive in 2 of 20 cases of hairy cell leukemia (Fig. 1D). PD-L1 staining was negative in all cases. All other B-cell neoplasms were negative for PD-1 and PD-L1 expression.

PD-1 expression is not well described on normal B cells, and in our experience with the use of immunohistochemistry and flow cytometry most naive/resting B cells lack PD-1 expression. When PD-1 is expressed by B-cell subsets, limited data suggest that PD-1 may be a negative regulator of B-cell activation and may have a role in the suppression of auto-reactive B cells by regulatory T cells^8^. In contrast, expression of PD-1 by neoplastic B cells of CLL/SLL and a subset of FL and diffuse large B-cell lymphoma is well documented^5^. In this report, we show that HCL expresses PD-1 at high levels and confirmed its co-expression on double-positive CD19 and CD25 neoplastic B cells by flow cytometry. There is a higher expression of PD-1 in HCL when compared to other low-grade B-cell lymphomas within the differential and similar expression when compared to non-neoplastic T cells. Although this finding was not consistently corroborated by immunohistochemistry this is most likely due to technical processing issues such as bone marrow biopsy decalcification. Future studies may be able to utilize clot sections for immunohistochemical staining purposes.

Firstly, this simple flow cytometry assay for PD-1 may be helpful in accurate diagnosis of HCL as virtually all cases of HCL express PD-1 when compared to normal B cells. Flow cytometric analysis is a vital tool in the diagnosis of hairy cell leukemia with few disease entities falling in its differential^9^. Splenic MZL, MZL, and HCLv cases among other low-grade B-cell lymphomas can confound the diagnosis as they have some overlapping clinical and immunophenotypic findings, but are treated differently^10,11^. It should be noted that possible variations in MFI may be encountered with different instrumentation, antibodies, and fluorochromes employed for analysis. In our study expression of PD-1 on HCL was uniformly high and at the level of background “exhausted” phenotype T cells. In T cells, the NFAT2 transcription factor is believed to be the critical transcriptional regulation of PD-1 expression^12^. In human metastatic melanoma cell lines, oncogenic BRAFV600E via MEK/ERK signaling leads to NFAT2 expression^13^. The genetic hallmark of HCL is BRAFV600E mutation (present in 30 of 32 of our cases) and NFAT2 is expressed by HCL^14^ suggesting that PD-1 expression may be a downstream effect of MAPK pathway activation. Whether PD-1 overexpression has a role in the pathogenesis of HCL or is just a bystander effect remains unclear. Interestingly, 3/32 cases were “negative” for PD-1 by our MFI analysis. Of these, 1 case was negative for BRAF and/or MAPK mutations, and 1 case had a concurrent myeloid neoplasm with a JAK2 V617F mutation.

Nevertheless, overexpression of PD-1 may have clinical implications. In addition to flagging cases that may or may not respond to BRAF inhibitors, therapies targeting the PD-1 pathway have been effective in B-cell lineage neoplasms overexpressing PD-1 such as CHL, a subset of diffuse large B-cell lymphomas, and Richter’s transformation of CLL/SLL^15^. Therefore, PD-1-targeted therapies may have a role in the management of HCL and these observations provide a strong rationale for investigating immunomodulating therapies in this setting.

In conclusion, flow cytometric results in our series show surface expression of PD-1 in hairy cell leukemia. PD-1 (CD279) is a readily available commercial probe and represents a useful tool to differentiate hairy cell leukemia from other small B-cell lymphomas in its differential, including HCLv. While PD-1/PD-L1 inhibitors have shown great promise in solid tumors including melanoma, non-small cell lung cancers, and more recently in Classical Hodgkin lymphoma, it is being studied with more vigor in hematologic malignancies and should be investigated in the context of hairy cell leukemia.

## Supplementary information

Supplemental material

Supplemental material
