# Supplementary material for: Hairy cell leukemia expresses programmed death-1
Source: Blood Cancer J. 2020 Nov 5;10(11):115. doi: 10.1038/s41408-020-00384-1 (PMC7644662; doi:10.1038/s41408-020-00384-1)

Title: Hairy Cell Leukemia Expresses Programmed Death- 1

Priyadarshini Kumar[1], Qi Gao[1], Alexander Chan[1], Natasha Lewis[1], Allison Sigler[1], Janine Pichardo[1], Wenbin Xiao[1], Mikhail Roshal [1], Ahmet Dogan [1]

Supplemental material

Supplemental Figure 1. ROC curve of CD279 MFI in Hairy cell Leukemia samples.

ROC curve of CD279 MFI in hairy cell leukemia samples shows high sensitivity and specificity in differentiating HCL from other B cell lymphomas ($p < 0.0001$, AUC 0.954: sensitivity of 75% and specificity of 98.5% using a threshold of 371.2).

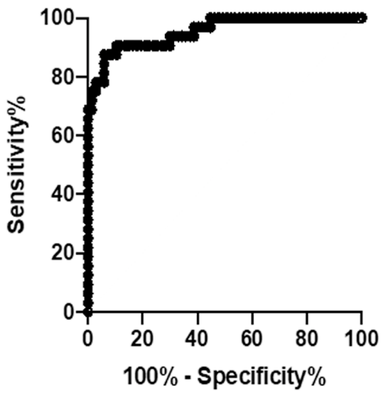

Supplement: Supplementary file 1 — Supplemental material [file 41408_2020_384_MOESM1_ESM.pdf]
