# Supplementary material for: Hairy cell leukemia expresses programmed death-1
Source: Blood Cancer J. 2020 Nov 5;10(11):115. doi: 10.1038/s41408-020-00384-1 (PMC7644662; doi:10.1038/s41408-020-00384-1)

Title: Hairy Cell Leukemia Expresses Programmed Death- 1

Priyadarshini Kumar^1^, Qi Gao^1^, Alexander Chan^1^, Natasha Lewis^1^, Allison Sigler^1^, Janine Pichardo^1^, Wenbin Xiao^1^, Mikhail Roshal ^1^, Ahmet Dogan ^1^

Supplemental material

**Supplemental Figure 1.** ROC curve of CD279 MFI in Hairy cell Leukemia samples.

ROC curve of CD279 MFI in hairy cell leukemia samples shows high sensitivity and specificity in differentiating HCL from other B cell lymphomas (p < 0.0001, AUC 0.954: sensitivity of 75% and specificity of 98.5% using a threshold of 371.2).


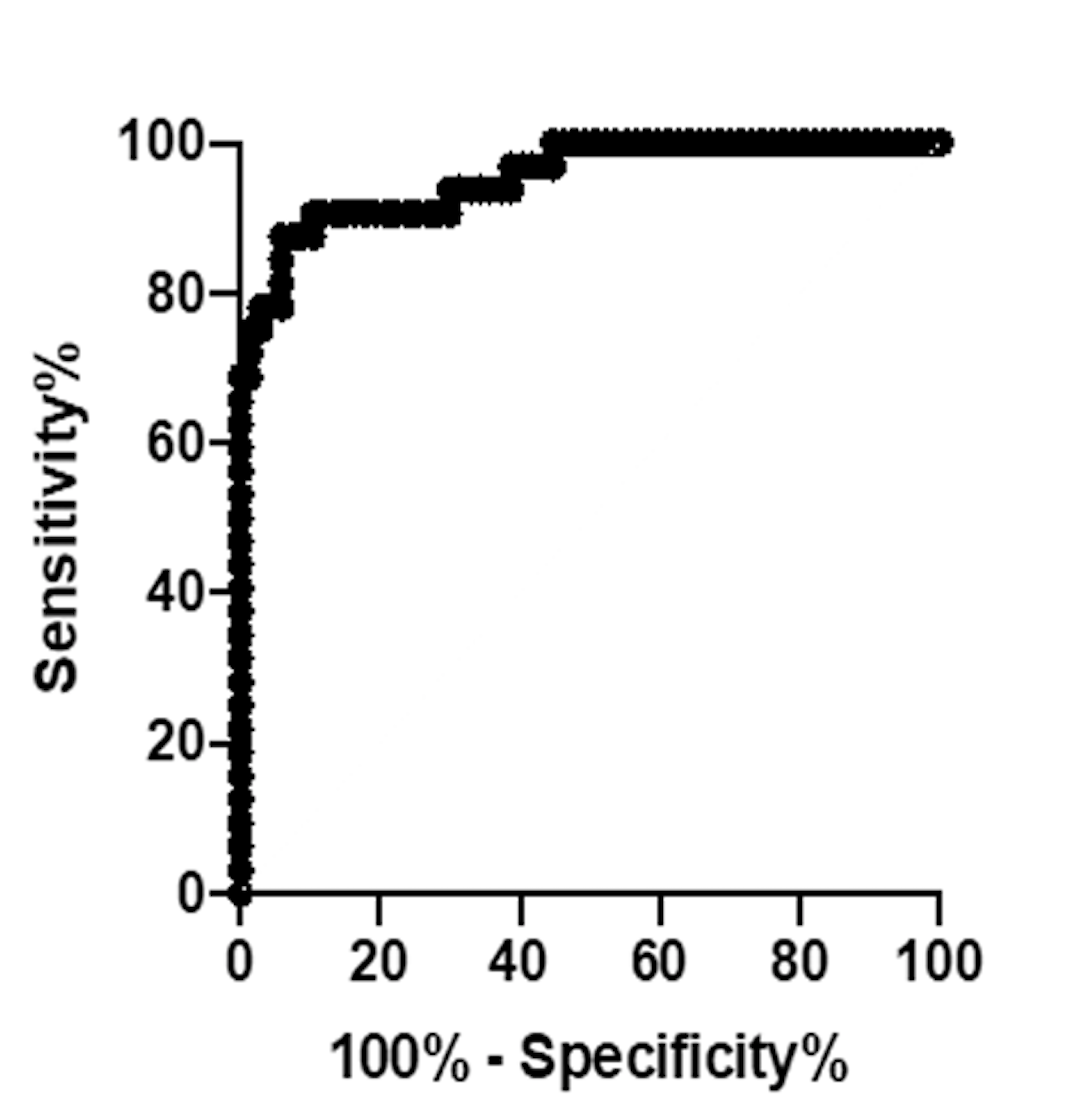

Supplement: Supplementary file 2 — Supplemental material [file 41408_2020_384_MOESM2_ESM.docx]
